# Late-onset neonatal sepsis in Suzhou, China

**DOI:** 10.1186/s12887-020-02103-y

**Published:** 2020-05-29

**Authors:** Tao Pan, Qiujiao Zhu, Pei Li, Jun Hua, Xing Feng

**Affiliations:** 1 Department of Neonatology, Children’s Hospital of Soochow University, Soochow University, Suzhou, China; 2 Department of Internal Medicine, Children’s Hospital of Wujiang District, Suzhou, China; 3 Department of Emergence, Children’s Hospital of Soochow University, Soochow University, Suzhou, China

**Keywords:** Late-onset sepsis, Neonate, Antimicrobial resistance, Mortality

## Abstract

**Background:**

This study aimed to describe the causative organisms of neonatal late-onset sepsis (LOS) and their antimicrobial resistance in Suzhou, Southeast China over a 7-year period.

**Methods:**

We performed a retrospective study on neonates with LOS from Jan1, 2011 to Dec 31, 2017. The demographic, clinical, and laboratory data of neonates with LOS were analyzed. Logistic regression was used to investigate the risk factors with mortality.

**Results:**

During the study period, 202 neonates with LOS were finally identified. The most common pathogens were *Escherichia coli* (29.2%), followed by *Klebsiella pneumoniae* (19.3%), and Coagulase-negative Staphylococcus (CoNS) (16.8%). Nearly 90% of the *K. pneumoniae* were resistant to cefazolin and 71.8% to ceftazidime. Thirty-four patients (16.8%) died. Multivariable logistic regression showed that significant predictors of mortality were birth weight < 1500 g, respiratory distress and convulsions.

**Conclusions:**

Gram-negative organisms have an important role in LOS in our region, with high levels of resistance to third-generation cephalosporins. These data may help in the selection of antibiotics for empirical treatment of neonates with sepsis.

## Background

Neonatal sepsis is an important complication among neonates, particularly those born prematurely [1–4]. The invasive infections can lead to severe morbidities and mortality, and increase the cost of medical care. In 2012, neonatal sepsis lead to 350,000 deaths in South Asia [1]. Neonatal sepsis increases globally because of the increase in premature infants [5, 6].. Managing neonatal sepsis is a challenge for physicians.

Depending on the onset age of the disease, neonatal sepsis is divided into early neonatal sepsis and late onset sepsis (LOS). Early neonatal sepsis is mainly due to organisms acquired before and during delivery, whereas LOS is due to organisms acquired after delivery from nosocomial or community sources. In the past few decades, the mortality rate of LOS has remained at a high level (5–15%) in most neonatal care facilities [7–9]. Coagulase-negative Staphylococcus (CoNS) is reported as the main pathogen of LOS [10–12]. However, the spectrum of pathogens varies among different regions and may change over time in the same place [13, 14]. The investigation of organisms causing LOS and the monitoring of their antimicrobial sensitivity can be used to guide appropriate empirical treatment and help develop cost effective interventions to prevent neonatal deaths.

China is known to be among the highest antibiotic consumers all over the world [15, 16]. However, there is no national wide survey about the pathogen distribution and their antimicrobial sensitivity in patients with LOS. This study aimed to describe the causative organisms of LOS and their antimicrobial resistance in Suzhou, Southeast China over a 7-year period. Risk factors with mortality were also investigated.

## Materials and methods

### Study design

We performed a retrospective study of LOS among neonates who were hospitalized in the neonatal intensive care unit (NICU) in the Children’s Hospital of Soochow University from Jan1, 2011 to Dec 31, 2017. The medical records of all neonates in the NICU were reviewed by two neonatologists. Children’s Hospital of Soochow University is a 1300-bed teaching hospital in southeast China that provides primary to tertiary care for children younger than 18 years old. This hospital has a NICU with a total of 50 beds. This study was approved by the Medical Ethics Committee of Children’s Hospital of Soochow University.

### Definition

LOS was defined as the growth of a single potentially pathogenic organism (bacterium or fungus) from blood or cerebrospinal fluid (CSF) in neonates > 3 days of age with clinical and laboratory findings consistent with infection [17]. Infants with only one positive blood culture for a common skin contaminant (e.g., diphtheroids, Bacillus, Propionibacterium, CoNS, or micrococci) without any symptoms of sepsis were excluded due to the possibility of contamination during sample handling. Blood and CSF Cultures and the identification of pathogens were performed using the VITEK®2 automated system (BioMérieux, France). Antibiotic susceptibility tests and the identification of ESBL were performed using antibiotic susceptibility test cards (VITEK®2 automated system).

### Data collections

Standardized forms were used to describe the maternal and neonatal characteristics for each infection case (antenatal screening cultures, mode of delivery, neonatal clinical signs and symptoms, age of onset, therapies, and outcomes).

### Statistics

Characteristics, clinical symptoms, and laboratory parameters of infants with LOS were described with data expressed as numerical values with percentage or mean and standard deviation as appropriate. Clinical symptoms were compared between preterm and term infants with LOS and characteristics compared between infants who died versus survived. Unadjusted comparisons were assessed by Fisher’s exact or chi-square test. A logistic regression model that included variables significant at *p* < 0.1 in univariate comparisons between infants who died and survived was used to examine adjusted associations with mortality. Adjusted odds ratios (OR) with 95% confidence intervals (CI) were reported from this model. Statistical analyses were performed using the Statistical Package for the Social Sciences (version 17.0).

## Results

### Demographics and clinical characteristics

During the study period, 5522 infants admitted to the NICU in Children’s Hospital of Soochow University were identified. Of these, a total of 213 neonates with suspected cases of sepsis were enrolled. Eleven cases were excluded because their cultures were considered contaminated. As a result, 202 neonates with LOS were finally identified. The demographic and clinical characteristics of the neonates are shown in Table 1. Of the patients, 84 (41.6%) cases were female and 118 (58.4%) were male. The mean birth weight was 2132 ± 719 g. The mean gestational age was 33.6 ± 4.2 weeks. The majority of infants with LOS were preterm (154/202, 76.2%) while about a quarter were term (48/202, 23.8%). Term infants present with clinical symptoms of sepsis at the time of admission or who developed sepsis during hospitalization were admitted to the NICU. The mean postnatal age at diagnosis was 13 ± 7 days. Fever (160/202, 79.2%), respiratory distress (118/202, 58.4%), neonatal jaundice (114/202, 56.4%) were the most common clinical manifestations. Respiratory distress (122 [79.2%] vs 25 [52.1%]) and feeding intolerance (115 [74.7%] vs 22 [45.8%]) were more common in preterm than in term neonates (both *P* < 0.01). There were no significant differences between preterm and term neonates in terms of fever (122 [79.2%] vs 38 [79.2%]), neonatal jaundice (102 [66.2%] vs 36 [75.0%]) and convulsions (16 [10.4%] vs 6 [12.5%], all *P* > 0.05).

**Table 1:** Demographic and Clinical Characteristics of the 202 Cases with Late Onset Sepsis

Characteristics	Number (%)
Sex	
Male	118 (58.4)
Female	84 (41.6)
Gestational age (wk)	
< 28	26 (12.9)
28–31	31 (15.3)
32–36	97 (48.0)
≥ 37	48 (23.8)
Birth weight (g)	
< 1500	48 (23.8)
1500- < 2500	93 (46.0)
2500- < 4000	41 (20.3)
≥ 4000	20 (9.9)
Cesarean delivery	125 (61.9)
Multiple gestation	9 (4.6)
Membrane rupture ≥18 h before delivery	69 (34.2)
Exposure to intrapartum antibiotics	53 (26.2)
Pregestational diabetes mellitus	21 (10.4)
Maternal intrapartum fever	32 (15.8)
Patients with underlying conditions ^a^	24 (11.9)
Clinical symptoms and signs	
Fever	160 (79.2)
Respiratory distress	118 (58.4)
Neonatal jaundice	114 (56.4)
Feeding intolerance	108 (53.5)
Convulsions	22 (10.9)
Laboratory parameters	
White blood cell counts (< 8 or > 12) 10^9^/L	166 (82.2)
Hemoglobin (< 110 or > 160) g/L	141 (69.8)
Platelet counts (< 100 or > 300) 10^9^/L	38 (18.8)
Procalcitonin (≥0.05) ng/mL	198 (98.0)
C-reactive protein (≥8) mg/L	153 (76.2)

^a^Among the 24 patients, 22 had congenital heart disease, 1 had congenital biliary atresia and 1 had intestinal duplication

### Etiologic pathogens

Among the 202 LOS, 86 (42.6%) episodes were caused by Gram-positive organisms; 104 (51.5%) episodes, by Gram-negative organisms; and 12 (5.9%) episodes, by *Candida albicans*. The most common pathogens were *Escherichia coli* (29.2%), followed by *Klebsiella pneumoniae* (19.3%), and CoNS (16.8%) (Table 2).

**Table 2:** The Microbiology Identified in 202 Neonatal Patients with Late Onset Sepsis in Suzhou, China

Pathogens	Total *n* (%)	Preterm *n* (%)	Term *n* (%)
Gram negative bacteria	104 (51.5)	83 (53.9)	21 (43.8)
*Escherichia coli*	59 (29.2)	49 (31.8)	10 (20.8)
*Klebsiella pneumoniae*	39 (19.3)	30 (19.5)	9 (18.8)
Klebsiella oxytoca	3 (1.5)	2 (1.3)	1 (2.1)
*Enterobacter aerogenes*	3 (1.5)	2 (1.3)	1 (2.1)
Gram positive bacteria	86 (42.6)	59 (38.3)	27 (56.3)
Coagulase-negative staphylococci	34 (16.8)	23 (14.9)	11 (22.9)
*Staphylococcus aureus*	17 (8.4)	10 (6.5)	7 (14.6)
Enterococcus faecalis	15 (7.4)	11 (7.1)	4 (8.3)
Group B Streptococcus	15 (7.4)	11 (7.1)	4 (8.3)
Listeria monocytogenes	5 (2.5)	4 (2.6)	1 (2.1)
Fungi	12 (5.9)	12 (5.9)	0
*Candida albicans*	12 (5.9)	12 (5.9)	0

Of all the 202 LOS cases, 149 (73.8%) received a lumbar puncture, and 32 (15.8%) of the CSF samples were positive. Of the 32 positive CSF cultures, five were from neonates with a negative blood culture. The other 27 infants have the same organisms isolated on both blood and CSF. *K. pneumoniae*, *E. coli*, and CoNS were the most common organisms isolated from the CSF of children with LOS.

**Fig. 1:**
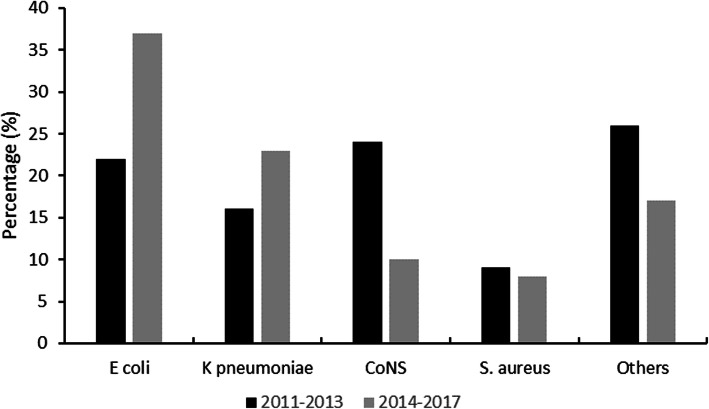
Distribution of the organisms of late onset infections between 2011 and 2013 and 2014–2017

We also compared the distribution of organisms between 2011 and 2013 and 2014–2017 (Fig. 1). During 2011–2013, CoNS was the most frequent cause of LOS (24.7%), followed by *E. coli* (22.7%) and *K. pneumoniae* (16.5%). In 2014–2017, *E. coli* was the most frequent cause of LOS (35.2%), followed by *K. pneumoniae* (21.9%) and CoNS (7.6%).

### Antibiotic susceptibility

All of the tested *E. coli* isolates (58/58) were sensitive to amikacin, nitrofurantoin, piperacillin/ tazobactam, cefoperazone/sulbactam, and imipenem (Table 3). Among the tested *K. pneumoniae* isolates, all (39/39) were sensitive to amikacin, piperacillin/tazobactam, cefoperazone/ sulbactam and imipenem, whereas nearly 90% were resistant to ampicillin (35/39) and cefazolin (34/39), 71.8% (28/39) to ceftazidime and 38.4% (15/39) to nitrofurantoin. Enterobacteriaceae isolates, including *E. coli, K. oxytoca,* and *K. pneumoniae* were also evaluated for extended-spectrum beta-lactamase (ESBL). Finally, 74.4% (29/39) of the *K. pneumoniae* isolates and 5.1% (3/59) of *E. coli* isolates were identified as ESBL Enterobacteriaceae. Among the tested ESBL Enterobacteriaceae isolates, all (32/32) were sensitive to piperacillin/tazobactam, cefoperazone/sulbactam; however, all were resistant to cefazolin and 78.1% (25/32) to ceftazidime. Among the tested CoNS isolates, all (34/34) were sensitive to vancomycin, 70.6% (24/34) were sensitive to cefoperazone/sulbactam, whereas 67.6% (23/34) were resistant to ampicillin and 61.8% (21/34) were resistant to cefazolin.

**Table 3:** Selected antimicrobial susceptibility patterns from the main bacteria of septic neonates, 2011 to 2017

Antibiotics	No. (%) susceptible		
	Coagulase-negative staphylococci (*n* = 34)	*Escherichia coli* (*n* = 58) ^a^	*Klebsiella pneumoniae* (*n* = 39)
Amoxicillin	11 (32.4)	–	–
Amikacin	–	58 (100)	39 (100)
Ampicillin	11 (32.4)	13 (22.4)	4 (10.3)
Cefazolin	13 (38.2)	26 (44.8)	5 (12.8)
Ceftazidime	22 (64.7)	49 (84.5)	11 (28.2)
Cefoperazone/sulbactam	24 (70.6)	58 (100)	39 (100)
Furadantin	34 (100)	58 (100)	23 (59.0)
Imipenem	–	58 (100)	39 (100)
Ciprofloxacin	27 (79.4)	25 (43.1)	33 (84.6)
Nitrofurantoin	34 (100)	58 (100)	24 (61.6)
Piperacillin/tazobactam	–	58 (100)	39 (100)
Sulfamethoxazole-trimethoprim	17 (50.0)	19 (32.8)	26 (66.7)
Vancomycin	34 (100)	–	–

^a^ Information on antibiotic sensitivity was available for 58 of the 59 neonates with *Escherichia coli* sepsis

### Infant antibiotic therapy

All of the neonates were treated with antibiotics. The majority of the neonates with infections (80.2%) were started on cephalosporins (first or third generation), while 18.8% received amoxicillin. After identifying the pathogen and its antibiotic susceptibility profile, antibiotics were changed for 43.6% of the neonates. Cefoperazone sulbactam, vancomycin and meropenem were the most frequently substituted antibiotics. Among the 86 neonates with Gram-positive bacterial infections, the majority, 65.1%, continued on cephalosporins or amoxicillin once the culture results were available, whereas 22.1% were switched to vancomycin with or without continuation of the initially-prescribed antibiotics. Among the 104 neonates who tested positive for Gram-negative organisms, 53.8% had their initial antibiotic therapies continued, whereas 37.5% were switched to meropenem or cefoperazone sulbactam.

### Mortality

Most neonates (83.2%) with LOS survived to hospital discharge, while 34 (16.8%) patients died; *E. coli* was responsible for nearly one-third (11/34, 32.4%) of deaths and CoNS responsible for 17.6% (6/34). The proportion of infants with birth weight < 1500 g was larger among infants who died compared to infants who survived (64.7% vs 15.5%, *p* < 0.001) as was the proportion born at gestational age < 32 weeks (55.9% vs 22.6%, p < 0.001) (Table 4). Respiratory distress, neonatal jaundice, and convulsions were also more frequently experienced by infants who died. Statistically significant differences between infants who died versus survived were not observed for the other characteristics examined, including the proportion with *E. coli* infection. When the 5 variables significant in univariate analysis were included in a logistic regression model, birth weight < 1500 g (OR:3.38; 95% CI: 1.33–8.62), respiratory distress (OR:4.25; 95% CI: 1.31–13.77), and convulsions (OR:4.09; 95% CI: 1.36–12.31) remained significantly associated with mortality (Table 5).

**Table 4:** Mortality Among Infants with Late-onset Sepsis

Characteristics	Died (n = 34), *n* (%)	Survived (*n* = 168), *n* (%)	*P* value
Birth weight < 1500 g	22 (64.7)	26 (15.5)	< .001
Gestational age < 32 wk	19 (55.9)	48 (22.6)	< .001
Male	19 (55.9)	99 (58.9)	0.74
Cesarean delivery	21 (61.7)	97 (57.7)	0.66
Multiple gestation	3 (6.8)	9 (3.4)	0.72
Pregestational diabetes mellitus	7 (15.9)	31 (11.8)	0.45
Maternal intrapartum fever	9 (20.1)	48 (18.3)	0.74
*E coli* infection	11 (32.4)	48 (28.8)	0.67
Clinical symptoms and signs			
Fever	24 (70.6)	136 (80.9)	0.18
Respiratory distress	30 (88.2)	88 (52.3)	0.001
Neonatal jaundice	12 (35.3)	102 (60.7)	0.01
Feeding intolerance	22 (64.7)	86 (51.2)	0.15
Convulsions	8 (23.5)	14 (8.3)	0.01

**Table 5:** Mortality Among Infants with Late-onset Sepsis

Characteristics	Died n/N (%)	*P* value	Adjusted OR for death (95% CI)
Birth weight < 1500 g			
Y	22/48 (45.8)	< 0.01	3.38 (1.33–8.62)
N	12/154 (7.8)		1.0
Respiratory distress			
Y	30/118 (25.4)	< 0.01	4.25 (1.31–13.77)
N	4/84 (4.8)		1.0
Convulsions			
Y	8/22 (36.4)	< 0.01	4.09 (1.36–12.31)
N	26/180 (14.4)		1.0
Gestational age < 32 wk			
Y	19/67 (28.4)	0.09	0.40 (0.14–1.17)
N	15/135 (11.1)		1.0
Neonatal jaundice			
Y	12/114 (10.5)	0.52	0.74 (0.30–1.84)
N	22/88 (25.0)		1.0

## Discussion

This study aimed to investigate the prevalence of culture-proven LOS in Suzhou, Southeast China and to identify the main causative organisms of LOS. Among 202 culture-proven LOS, *E. coli* was the most commonly isolated bacteria, followed by *K. pneumoniae* and CoNS.

Previous studies have revealed that gram-positive bacteria were the main causative organisms, with CoNS responsible for the most common organism of LOS [18, 19]. Majeda et al. reported that CoNS alone was responsible for more than one-third of LOS cases in Arab states in the Gulf region [20]. Matthew et al. found that for nearly 20 years, CoNS was the organism most responsible for late-onset sepsis in their NICU [21]. In some regions from developing countries, CoNS have been reported to play a less important role [22, 23]. In China, Guo et al. found that CoNS was the third common organism responsible for LOS in South China [24], while Li et al. found that CoNS was the most common organism in Southeast China [25]. The results in our study are in line with Guo’s study. Thus, the role of CoNS responsible for LOS are likely to vary in different regions in China. Despite the importance of CoNS as etiological agents of LOS, determining whether CoNS isolates are true pathogens or contaminants is still very challenging [21, 26]. In our study, CoNS were considered pathogenic only if, in addition to a positive blood culture, there were clinical and laboratory findings to support infection. Furthermore, 6 died from LOS by CoNS, suggesting that these were true infections.

*E. coli* and *K. pneumoniae* were the most common isolated Gram-negative bacteria in our study, accounting for 29.2 and 19.3% of all the isolated pathogens, respectively. Other Gram-negative bacilli were recovered but in a few numbers. The predominance of *E. coli* and *K. pneumoniae* among the causative Gram-negative pathogens was also reported in other studies in China and other different countries [24, 27]. In contrast, Enterobacter was reported as the most common Gram-negative isolates in other studies [20, 28].

Fungi are also significant pathogens of neonatal sepsis, especially in preterm and low birth weight infants. Fungi infections are usually acquired during prolonged hospital stay of preterm neonates [29]. In our study, Candida spp. accounted for 5.9% of isolated pathogens. All the patients with Candida spp. infection were preterm low birth weight infants. The fungi infection rate in our study is similar to the results of Guo’s study [24], but significantly higher than the results of 2 other studies conducted in other regions of China [13, 14].

In our study, more than 70% of the patients received lumbar puncture. and 15.8% of the CSF samples were positive. Neonatal meningitis remains a substantial cause of sepsis-related morbidity and mortality in neonates. Other than CSF cultures, there are no clinical parameters that excludes the diagnosis of meningitis in neonates [30]. Hoque et al. also reported that it is important to do a lumbar puncture for all suspected septicemia cases in neonates. They found that the clinical manifestations were similar in both sepsis and meningitis cases, while mortality was high among the meningitis cases compared with those having sepsis alone (37.5% vs. 13.3%) [31].

Because LOS is usually caused by nosocomially acquired organisms, it has been suggested that resistance to antibiotics is common in LOS pathogens. As the second common pathogen responsible for LOS, nearly 90% were resistant to cefazolin, 71.8% to ceftazidime. The high level of resistance of *K. pneumoniae* to third-generation cephalosporins has been reported from developing countries [23, 32]. Among the tested CoNS isolates, all were sensitive to vancomycin. Vancomycin is the most effective and economical drug for treatment of CoNS LOS. However, great concern has been raised due to the widely used vancomycin and the emergence of vancomycin-resistant *Staphylococcus* [33, 34]. Actually, in our study, about 70% of the CoNS isolates were sensitive to cefoperazone/sulbactam. We suggested that vancomycin use should not be empirical but based on the results of blood cultures in our region. In China, there are currently no national antimicrobial guidelines for neonatal sepsis. The development of guidelines could be assisted by the findings of our study on both epidemiology and antimicrobial use. Local epidemiology and antimicrobial susceptibility patterns might explain the absence of national guidelines. In our region, the current resistance profile of pathogens makes empiric coverage with cefoperazone/sulbactam seem appropriate.

Previous studies reported that appropriate empiric treatment significantly reduced the duration of treatment [26]. A study by Chiu et al. also showed that appropriate empiric treatment of suspected CoNS LOS significantly reduced the use of vancomycin [35]. As reported by Kaufman, accuracy in diagnosis and use of antimicrobial agents are important to prevent neonatal mortality related to sepsis [36]. In our study, 65.1% of the neonates who tested positive for Gram-positive organisms and 53.8% of the neonates who tested positive for Gram-negative organisms had their initial antibiotic therapies continued. Whether appropriate empiric treatment reduced the duration of treatment in our region need further investigations.

In our study, The case-fatality was 16.8%, which is similar to those reported from Asian countries (16%) [10, 20]. *E. coli* were responsible for about one-third (11/34) of deaths due to LOS. *E coli* has been found to be associated with most LOS deaths, primarily because of its predominance among very low birth weight infants [21]. However, in our cohort of mostly preterm infants *E. coli* infection was not an independent predictor of mortality. We found that significant predictors of mortality were birth weight < 1500 g, respiratory distress and convulsions. Thus, clinicians should pay more attentions to the low birth weight infants presented with respiratory distress and convulsions.

In conclusion, Gram-negative organisms have an important role in LOS in our region, with high levels of resistance to third-generation cephalosporins. These data may help in the selection of antibiotics for empirical treatment of neonates with sepsis.

## Data Availability

The manuscript detailing where the data supporting the findings in this study can be found if requested.

## Abbreviations

LOS: Late onset sepsis
CoNS: Coagulase-negative Staphylococcus
CSF: Cerebrospinal fluid
ESBL: Extended-spectrum beta-lactamase
